# Production of Sustainable and Biodegradable Polymers from Agricultural Waste

**DOI:** 10.3390/polym12051127

**Published:** 2020-05-14

**Authors:** Chrysanthos Maraveas

**Affiliations:** Department of Civil Engineering, University of Patras, 26500 Patra, Greece; c.maraveas@maraveas.gr

**Keywords:** polymers, sustainability, biodegradable polymers, agricultural waste, cellulose, reinforcement, biofilms, tensile strength, photo degradation, water permeability, food packaging

## Abstract

Agro-wastes are derived from diverse sources including grape pomace, tomato pomace, pineapple, orange, and lemon peels, sugarcane bagasse, rice husks, wheat straw, and palm oil fibers, among other affordable and commonly available materials. The carbon-rich precursors are used in the production bio-based polymers through microbial, biopolymer blending, and chemical methods. The Food and Agriculture Organization (FAO) estimates that 20–30% of fruits and vegetables are discarded as waste during post-harvest handling. The development of bio-based polymers is essential, considering the scale of global environmental pollution that is directly linked to the production of synthetic plastics such as polypropylene (PP) and polyethylene (PET). Globally, 400 million tons of synthetic plastics are produced each year, and less than 9% are recycled. The optical, mechanical, and chemical properties such as ultraviolet (UV) absorbance, tensile strength, and water permeability are influenced by the synthetic route. The production of bio-based polymers from renewable sources and microbial synthesis are scalable, facile, and pose a minimal impact on the environment compared to chemical synthesis methods that rely on alkali and acid treatment or co-polymer blending. Despite the development of advanced synthetic methods and the application of biofilms in smart/intelligent food packaging, construction, exclusion nets, and medicine, commercial production is limited by cost, the economics of production, useful life, and biodegradation concerns, and the availability of adequate agro-wastes. New and cost-effective production techniques are critical to facilitate the commercial production of bio-based polymers and the replacement of synthetic polymers.

## 1. Introduction

This review article explores the production of biopolymers, biodegradable polymers, and polymers from agricultural waste such as fruit seeds, fruit peels, coconut shells, potato peels [1], orange tree pruning [2], wheat straw [3], soy protein isolates [4], oil palm fiber [5], sugar palm, corn starch, and rice husks, which are categorized as renewable sources. This review’s purpose is to provide conclusive evidence on whether biopolymers, biodegradable polymers, and polymers from agricultural waste were fully biodegradable or only compostable. Current experimental data show that polymers compost at different rates in the environment [6,7].

The investigation of green materials such as bio-based plastics is validated by the contribution of synthetic plastics materials to anthropogenic contamination of the environment in each phase of the life cycle—from monomer synthesis to disposal in landfills or recycling [8]. The current rate of global plastic production is unsustainable, considering more than 400 million tons of waste are generated each year. Additionally, the rate is expected to increase fourfold by 2050 [9] and there has been a concomitant increase in agricultural plastic waste [10]. The agricultural plastic waste originates from shading nets, mulching materials, and pesticide containers. The volume of agricultural plastic waste would surge in line with the global demand for food cultivated in controlled environments [11]. The quantity of agricultural waste derived from various supply chains was about 90 million tons of oil equivalent (MTOE) [12]. Considering that only a small fraction of the waste is utilized in the production of animal feeds, manure, and other value-added products, there is a potential for the production of biodegradable polymers from agricultural waste. The recycling of plastic waste is not favorable using current technologies due to the risk of leakage of toxic and synthetic chemicals such as anti-oxidants, plasticizers, and stabilizers [13]. The absence of facile, scalable, and environmentally favorable recycling processes has impacted the rate of recycling of global plastics waste—only 9% of the plastics are recycled [8,9]. The threat of plastics to the environment extends beyond the lack of suitable recycling methods; the synthesis of eco-friendly polymer composites has been impacted by unsuitable synthetic routes.

The rate of non-biodegradable plastic production and landfilling coupled with the rapid growth in the global population show that the traditional model, which was primarily based on the extraction of raw materials, production, use, and disposal, is no longer viable in the 21st century and beyond. Environmental advocates have championed the adoption of a new approach to manufacturing that ensures that today’s usable products create resources and materials for the development of tomorrow’s products [14]; this can be achieved through a circular modern business typology, which integrates agricultural cooperatives, agro-parks, support structures, environmental biorefineries, upcycling entrepreneurship and biogas plants [14].

Beyond the circular business model typology, sustainability can be enhanced through the production of biodegradable polymers. The current state of research on the production of biodegradable polymers has adopted two approaches. One, biodegradable polymers are manufactured from bio-based precursors, such as agricultural waste, starch [15], and renewable materials such as poly(lactic acid) (PLA) and polyhydroxyalkanoates (PHA), which are produced by Gram-positive and Gram-negative bacteria [7,16]. Two, the bio-based polymers are synthesized through the modification of non-biodegradable polymers. The microstructure of non-biodegradable polymers can be modified through the integration of anti-oxidants [4] and pro-oxidant additives, which induce photo-oxidation and oxo-degradation following exposure to ultraviolet light. The utility of the second approach in achieving 100% biodegradation has been contested because non-biodegradable polymers are infused with synthetic stabilizers and photo-initiators, which act as inhibitors in the biodegradation process and UV-oxidation. Considering the limitations of the latter method, the scope of this review is confined to the synthesis of bio-based polymers from renewable sources, especially agricultural wastes. The sources of agricultural waste include post-harvest waste from horticultural plants [8], sugarcane bagasse [3], rice husks, and bamboo leave ash [17].

## 2. Production of Biodegradable Polymers

**Figure 1:**
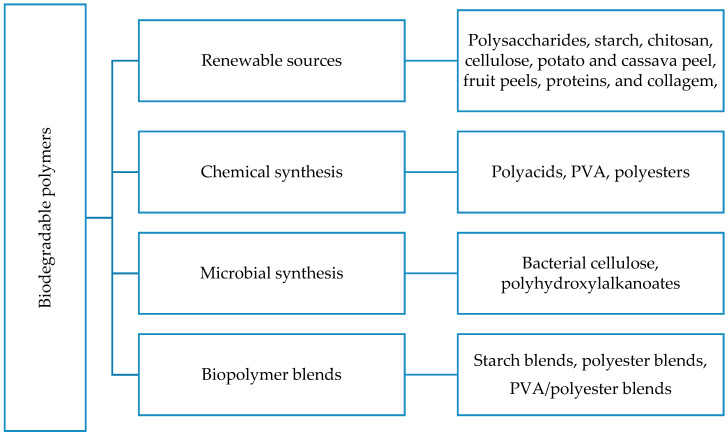
Classification of production processes for biodegradable polymers [18] (reproduced with permission from publisher).

Biodegradable polymers are a unique class of polymers that are ecologically benign (biocompatible and biodegradable), as shown in Figure 1. The production process for these biopolymers is grouped into four different classifications depending on the desired products and the available materials/precursors. The classifications are chemical synthesis methods, bacterial synthesis methods, biopolymer blends, and renewable sources [18]. The present discussion primarily focuses on the first type of production, which focuses on the production of bio-based polymers from agricultural waste. Other synthetic routes (biopolymer blends, chemical, and bacterial synthesis) are discussed briefly in the subsequent sections.

The selection of the biodegradable polymers for various commercial applications is based on the physical properties of the polymers. High tensile strength, tensile strength, and yield strength are critical in construction-related applications. In contrast, the % elongation determines utility in packaging. Following the review of the information presented in Table 1, poly(glycolic acid) (PGA) biopolymer has the best tensile strength and modulus of elasticity, but a lower percentage elongation at break [18]. The data show that the mechanical strength is correlated with the density; a higher density translates to better mechanical strength.

### 2.1. Production of Bio-Based Polymers from Renewable Sources and Agro-Wastes

The production of bio-based polymers from agro-wastes is influenced by the availability of starting materials/precursors; these materials should be cheap and available in significant quantities. Among the leading economies, India and China have the capacity to lead in the production of fruit and vegetable-based biopolymers, given the high production capacity and share of total global production [19]. The renewable sources for bio-based polymers are diverse. Bio-based polymers have been synthesized from plant-based precursors containing lignocellulose fibers, cellulose esters, polylactic acid, and polyhydroxyalkanoates (PHA) [11]. The lignocellulosic fibers are derived from plants such as curaua, pineapple, sisal, and jute [18]. The physical properties of the final product are largely determined by the extraction method. Organic materials/precursors containing large quantities of cellulose and other fibers are preferred because they enhance the mechanical strength of the materials. On the downside, even though cellulose is a bio-based material, the precursor is non-biodegradable due to the higher degree of substitution [4]. In contrast to other renewables, which are sourced from plants, agricultural waste comprises of post-harvest waste, by-products of food processing such as coconut shells [20], potato peels [1], fruit peels [21], and fruit seeds [22], which have been traditionally discarded as waste in farms and food processing facilities.

Agricultural waste is a primary source of starting materials, which are used in the production of bio-based plastics, plasticizers, and antioxidant additives [1]. Vegetable-based agricultural wastes are a vital source of polysaccharides, which are essential precursors in the development of natural plasticizers [23]. The main function of the plasticizers is to enhance the elasticity and mechanical strength of the bio-based polymers. The performance of vegetable-derived polysaccharide plasticizers relative to glycerol and other synthetic plasticizers has not been determined [22], and commercial application is limited. Agricultural waste such as mango kernel extracts, green tea extracts, essential oils, proto-catechuic acid, grapefruit seed extract, and curcumin sourced from food processing facilities are used in the development of antioxidant additives [1]. Other agro-wastes that are viable sources of natural antioxidants include pomegranate peel extract (PE), mint plant extracts (ME) [21], *Thymus vulgaris L*. and oregano [24]. The phenols in the natural antioxidants are Lewis bases and electron donors, which are critical to the anti-oxidation activities. Apart from phenols, pomegranates contain gallic acid and gallates, which are natural stabilizers and indicators of aging [25].

Bashir, Jabeen, Gull, Islam, and Sultan (2018) noted that these materials had the prerequisite antioxidant activity that was linked to the ability to scavenge for OH groups and oxygen radicals in 2,2-diphenyl-1-picryl-hydrazyl-hydrate (DPPH). The performance of these materials is comparable to synthetic antioxidants and could, therefore, replace existing additives such as the carcinogenic butylated hydroxytoluene [21]. The main challenge is that the performance of the natural additives on a commercial scale has not been confirmed. The main function of the additives is to inhibit the UV-based photodegradation of the plastics following exposure to sunlight [26]. However, the utilization of natural additives is a new phenomenon; commercially available bio-based plastics have incorporated synthetic additives. Apart from the incorporation of natural additives, UV-induced degradation is inhibited by maleic anhydride treatment, direct, reactive mixing, and graft copolymerization during synthesis [27].

The utilization of waste from renewable sources for commercial purposes has the potential to reduce the rates of global warming, considering that compositing and landfilling contribute to global warming. Data collected from Italy show that the recycling of agricultural waste through composting and the production of fertilizers increases global carbon emissions. In particular, 64 and 67 kg of CO_2_ equivalent was generated per mg from olive waste-based compost (OWC) and anaerobic digester-based compost (AD), respectively. Additionally, re-composting and co-composting generated between 8 and 31 kg of CO_2_ per mg of compost [28]. The data obtained from the composting experiments show that recycling of agricultural waste poses a threat to the environment, and it is not ecologically beneficial as initially proposed. The significant quantities of CO_2_ equivalent emissions generated per mg of compost indicate that novel methods of utilizing agricultural waste such as the production of bio-based polymers are necessary; this because the latter methods are more sustainable and have a lower ecological impact based on the LCA analyses.

Global statistics show that the production of bio-based plastics from renewable sources was low—2.1 million tons were produced in 2018 [13]. The projected demand for bio-based polymers would be equivalent to 46% of the global production of packaging plastics by the end of 2020 [29]; this translates to about 7 million tons [29]. The demand for bio-based plastics in food packaging is based on the unique material properties of biofilms relative to synthetic alternatives. The bio-based polymers absorb ethylene, remove water vapor, protect fruits and vegetables from microbial contamination due to the presence of anti-microbial agents [30], protect against UV radiation, and are easily recyclable [31]. Current bio-based polymers have shown effective antimicrobial performance against *Bacillus subtilis, Escherichia coli,* and *Listeria monocytogenes* [32]. The bio-based films have other essential properties that influence the development of intelligent packaging systems [33].

The ability of current production systems to satisfy this demand is unknown, considering that nearly 50% of the bio-based plastics made from renewable feedstock were non-biodegradable, possibly due to the addition of synthetic plasticizers, and other additives to enhance their mechanical properties. The leading synthetic plasticizers include polyethylene glycol, citrate ester, and oligomeric acid [4]. Rameshkumar, Shaiju, Connor and Babu (2020) [34] noted that global estimates are not entirely accurate due to the complexity of the supply chains, continuous innovation, and commercial release of new varieties of bio-based polymers. The data show that there were two inherent challenges associated with the production of bio-based polymers. Firstly, the production capacity is low, and it cannot match the production of non-renewable plastics, whose production was estimated at 400 million tons [10]. Secondly, current technologies are limited and inadequate—there are no 100% biodegradable bio-based polymers with optimal mechanical properties. Other challenges are discussed in Section 3.

Considering the global variability in the availability of agricultural waste, the development of the materials would be concentrated in specific geographical areas. For example, fruit peels and coconut shells are found in abundance in tropical and coastal areas, respectively [20]. Since India and China have a high fruit and vegetable production capacity [19], agro-wastes synthesized from fruit and vegetable wastes would be abundant in Asia. Coconut shells and microalgae are abundant in coastal areas and marine environments, respectively [20,35]. Jackfruits and other similar plants grow best in tropical and subtropical climates [20]. The data show that the production of bio-based plastics from agro-waste should be customized to suit the available precursors. The development of bio-based polymers from locally available agricultural wastes would also help to reduce the carbon footprint.

Polymers that are made of poly(butylene adipate-co-terephthalate), poly(butylene succinate/adipate), and poly(e-caprolactone) are biodegradable because the carbon chains are susceptible to enzymatic degradation [12]. Commercially available biopolymers are grouped into the following categories: polylactides (PLA), polyhydroxyalkanoates (PHAs/PHBs), polyols, polyamides, bio-PET, butyl rubber, and cellulose acetate [36]. PHAs are further grouped into long-chain, medium, and short-chain polymers [16]. The length of the chains predicts the utility in commercial applications; short-chain polymers are not ideal in high strength applications owing to their brittleness, high degree of crystallinity, and stiffness. Medium chains are less susceptible to brittle fracturing owing to the high elastic modulus, flexibility (longer elongation at break), and low crystallinity. However, the materials are less suitable for high-temperature applications [16].

The selection of suitable agro-waste is based on the following primary criteria: (i) starch content; (ii) cellulose and lignin and hemicellulose content (iii) bioavailability and impact on agricultural supply chains and food security (iv) complexity of the synthetic routes and desired material properties; (v) biodegradation [20,35,37,38]. Based on the data presented in Table 2, corn and stalks have the highest cellulose concentration % w/w, which is critical for high strength applications. Experimental data indicate that the production of biopolymers involves a tradeoff between the cellulose content and the rate of biodegradation—plant cellulose limits the rate of biodegradation but enhances the mechanical strength of the polymer films—a challenge that has been resolved by Xie, Niu, Yang, Fan, Shi, Ullah, Feng, and Chen [1]. The study reported the successful replacement of plant cellulose with bacterial cellulose [1]. The cellulose and starch content are limiting factors in the selection of agricultural waste precursors.

Bio-based polymers synthesized from different agro-wastes have distinct material properties. The starch content in the agro-waste predicts the thickness of the bio-based plastic films—higher starch content is correlated with an optimal thickness (~0.099–0.1599 mm) due to the presence of amylose and amylopectin compounds [22]. Thick films have better mechanical properties compared to thin films. For example, *Chlamydomonas reinhardtii* microalgae species yield the highest starch content after 800 h of inoculation [35]. Based on the inoculation experiments, *Chlamydomonas reinhardtii microalgae* species would be highly preferred as precursors in the development of bio-based polymers compared to other species such as *Scenedesmus sp* and *Chlorella variabilis.* Starch content is one of the primary criteria in the selection of the agricultural precursor. The preference for species with a high starch content involves a tradeoff with the rate of culture growth. Similarly, a higher cellulose content augments the mechanical strength but limits the rate of biodegradation [11,40].

#### 2.1.1. Thermoplastic Starch-Based Polymers

Starch is a polysaccharide found in tubers, legumes, and cereals agro-wastes and is an ideal carbon precursor for bio-based polymers [41]. Thermoplastic starch-based polymers are practical alternatives to petroleum polymers based due to effective reinforcement properties, abundance, and tunable properties [38]. The base material, starch (derived from potatoes, cereals, and corn), is abundant in the biosphere [35] and it has been extensively explored in research, as noted by Tabasum, Younas, Ansab, Majeed, Majeed, Noreen, Naeem, and Mahmood [42]. The first phase in the production of starch-based polymers from agro-wastes involves the addition of L-lactate and a catalyst (Sn(oct)_2_). Alternatively, the polymerization process can be triggered by the addition of the poly 1,4-dioxan-2-one (PPDO)–diisocyanate (NCO) group, leading to the formation of starch-g-PPDO polymer chains [18]. Although the process is scalable, the PPDO–NCO + starch/Starch + L-lactide and Sn(Oct)_2_ reaction results in biopolymers that are easily degraded by water—the addition of plasticizers limits susceptibility to water degradation. The yield (Y%) and grafting efficiency (GE) of the starch-PPDO and NCO synthetic route are determined using the formula depicted in Equations (1) and (2), respectively [43]. W1 denotes the starting weight and final weight. The main challenge with this synthetic route is eco-toxicity. The production of PPDO–NCO relies on 2, 4-Tolylene diisocyanate, and other chemicals that have been proven as toxic to the human body. The use of toxic chemicals impacts the cradle-use-disposal cycle.

(1) $$Y\%=\frac{W_{1}*100}{W}$$

(2) $$GE\%=\frac{\left(W_{1}-W_{2}\right)*100}{\left(W-W_{2}\right)}\;\;$$

Current research has shown that these materials are critical to the future of sustainable food packaging because they are flexible and light [34]. Commercial application is limited by poor water resistance, poor mechanical strength, and risk of dissolution in water—a challenge that is addressed by blending with other polymers to enhance the mechanical strength. Alternatively, TS materials are reinforced by the incorporation of ionic liquids such as 1-butyl-3-methylimidazolium chloride in the pretreatment process and the production of bio-composites [44]. The surface treatment process results in the development of materials with greater activation energies, which predicted the rates of thermal degradation. Biopolymers with cellulose are degraded at temperatures of up to 500 °C [45]. The rate of thermal degradation influences end of life treatment and application in high-temperature applications. Other constraints include complex synthetic processes such as plasticizing, casting, and extrusion, which are difficult to replicate on a commercial scale.

The material property challenges associated with the starch-based polymers are dependent on the starch precursor. Sugar palm, microalgae, and jack fruit result in starch-based polymers with distinct properties [22,35,37], and the synthetic route should be customized to suit the polymer applications. The natural properties of bio-based polymers are modified through the addition of tetraethoxysilane (TEOS), polyvinyl alcohol (PVA), and chitosan. The PVA is used to enhance mechanical properties [46]—a higher PVA ratio compared to the filler was correlated with greater mechanical strength. However, the chemicals (borax and formaldehyde) used in the chemical cross-linking of the biopolymers are toxic and non-biodegradable [21]. Chitosan helps to improve the bonding between natural polymers, TEOS, and PVA [47]. Apart from the material constraints and complex synthetic routes, the sustainability of starch-based polymers is questionable on a commercial scale because starch sources are staple foods in most countries. From a food security perspective, large-scale commercial production of thermoplastics might be a threat to food security. The challenges and viable alternatives in the commercialization of biodegradable polymers are discussed in the next section.

#### 2.1.2. Production of Bio-Based Plastics from Pineapple Peels and Tomato Pomace

The production process of bio-based polymers from pineapple peel is based on a standard method that involves the extraction of biopolymers from agricultural waste. The initial procedures involve the analysis of the chemical composition, especially the C/N and C/P ratios, which predict the polymer yields [48]. Once the number of trace metals, ash, and carbohydrates, protein, the peels are fermented (using dipotassium phosphate or ammonium sulfate) and subsequently hydrolyzed with H_2_SO_4_, the biopolymers are extracted via centrifugation at a rate of 4000 rpm or higher. FTIR, NMR, and GC-MS instruments are used in the characterization of the final product. The information presented in Table 2 shows that optimal PHA yields were reported in precursor materials that had the highest content of C/N and C/P. Additionally, the biopolymer yield is influenced by time and pH optimization. The optimal time and pH were 60 h and 9, respectively [48]. The yield data show that chemically induced fermentation was capable of complementing natural bacterial synthesis methods. The only constraint is the possible adverse effect of synthetic chemicals such as H_2_SO_4_ and dipotassium phosphate or ammonium sulfate, among other chemicals, which may potentially contribute to acidification and eutrophication [49] in the environment if used in large quantities.

**Figure 2:**
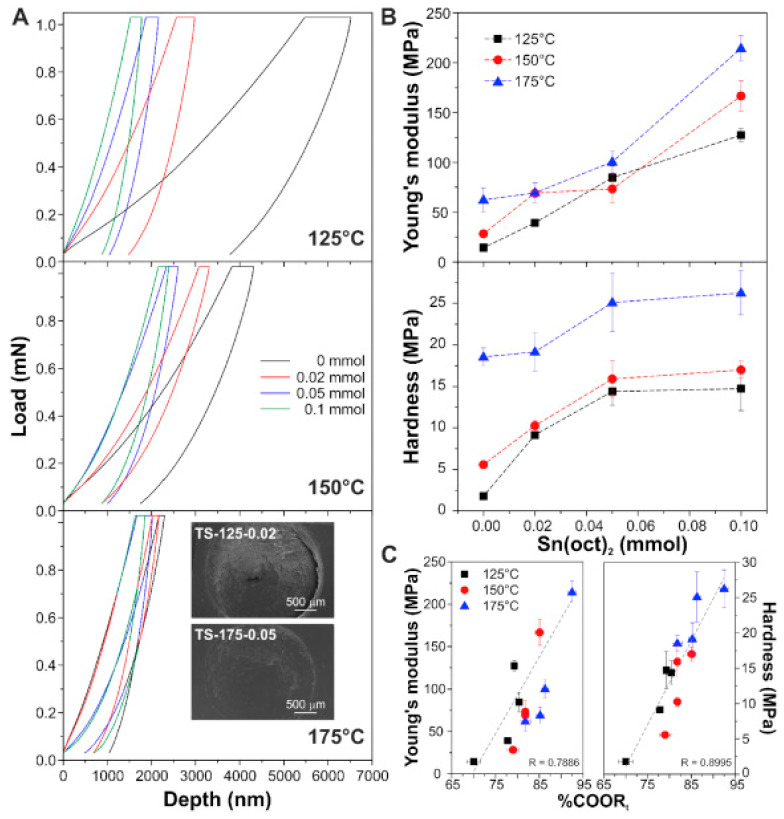
Mechanical behavior of biopolymers synthesized from tomato pomace [8]; (**A**) the load-depth indentation curves for biopolymers that were synthesized with different amounts of catalysts, for 7 h; (**B**) impact of catalyst amount on the Brinnell hardness and Young’s modulus; (**C**) shows the linear regression relationship between % ester in the polymer, Young’s Modulus and hardness. (Reproduced with permission from publisher).

The production of bio-based polymers from tomato pomace follows a similar approach as the production of bio-based polymers from the pineapple peels [8,48], except for the melting poly-condensation step. The mechanical properties of biopolymers derived from tomato pomace are presented in Figure 2A. Since the linear regression values are close to 1, the linear regression graph in Figure 2C confirms that the formation of ester functional groups (COOR-) influenced the hardness and Young’s modulus of the biopolymer. The data show that optimal mechanical properties were achieved at 175 °C. The volume of the catalyst (Sn(oct)_2_) impacted the depth of the indent caused by the Brinnell hardness, as shown in Figure 2. Optimal depth was reported in samples with 0.00 mmol of the catalyst. A contrary phenomenon was noted in the relationship between catalyst (Sn(oct)_2_), Young’s modulus, and hardness.

Apart from the production of bio-based polymers, fruit peels are effective in enhancing the mechanical properties of manufactured polymers. Patil, Hrishikesh and Basavaraj [50] observed that the addition of 10–30% of lemon peel powder and sweet lime peel powder reinforces the mechanical strength of natural fibers and epoxy resins [50]. Optimal mechanical performance (Brinnell hardness of 83, the flexural strength of 79 MPa, and tensile strength of 48 Mpa) was reported in the epoxy-lemon biopolymer, which had a 30% volume weight of lemon particles. The improvement in the mechanical properties was linked to the presence of cellulose, lignin, and crude fibers, which made up of 90% of the sweet lime and lemon [50]. Additionally, there was good particle distribution and particle-matrix adhesion. Even though the sweet lime and lemon peel showed ideal properties in the reinforcement of the structures, the sustainability aspect remains a challenge; this is because the lemon and sweet lime fruits are edible and the commercial availability of waste fruit peels is not guaranteed. In advanced markets, the fruit peels are used to produce value-added products such as bioactive polyphenols [50]. Phenol-containing compounds have natural antioxidant capabilities [21]. Alternatively, the peels are ingredients in the manufacturing of home-based beauty products. The production of lactic acid and poly-lactic acid from agro-wastes is discussed in the next section.

#### 2.1.3. Production of Lactic Acid, PLA and PHA from Agro-Wastes

The production of lactic acid and poly-lactic acid [51] is influenced by specific strains of bacteria for fermentation and hydrolysis and the availability of agro-wastes as starting materials [51]. The fungi and bacteria strains adopted for commercial applications include *Rhizopus, Pediococcus*, and *Streptococcus* [51]. The availability of a wide array of bacteria and fungi species has an impact on the material properties (biochemical characteristics, morphological, and psychological characteristics) of the final product due to the variations in the fermentation processes that lead to the production of fermentable sugars such as starch and cellulose. Apart from the utilization of different strains of bacteria, the material properties of the PLA- and lactic acid-based polymers are influenced by the pre-treatment methods (cold and thermal) that are primarily used to remove undesired materials. The fermentation process results in the formation of lactic acid, which is polymerized to form PLA. Biopolymers that are synthesized from agricultural wastes have a tensile strength of 36.3 MPa and a melting point of 170 °C. The high tensile strength and melting point show that the polymers are suitable for packaging applications and agricultural shading.

#### 2.1.4. Production of Bio-Composites from Winery Agro-Wastes and Sugar Beet

Merlot grape pomace fruit waste is the main form of winery agro-waste. In place of decomposition, the winery agro-wastes are a suitable source of composites that are manufactured through solvent extraction (SE) methods, and pressurized liquid extraction (PLE). The extracts drawn from PLE and SE methods are mixed with commercial-grade polyhydroxyalkanoate to form the matrix. The final phase of the production involves mixing the biopolymer with the poly(3-hydroxybutyrateco-3-hydroxy valerate) (PHBV)—a copolyester containing hydroxyaleric acid to form active bio-composites [52]. The bio-composites have higher or higher than normal tensile strength compared to the virgin biopolymers or the matrix in isolation. The data presented in Table 3 and Table 4 show that the highest mechanical strength was reported in the virgin PHBV matrix. The inclusion of the bio-based materials extracted via solvent extraction resulted in a reduction in the tensile strength and a marginal improvement in the elongation at break. The data also show that the synthetic route/extraction method for phenols had an impact on the mechanical properties of the bio-composites—solvent extraction (SE) was a practical solution compared to pressurized liquid extraction (PLE) [52].

Beyond grape pomace, sugar beet agro-wastes are practical sources of bio-composites owing to the presence of carbocal in the dried pulp [53]. The mechanical properties of the Carbocal are enhanced through the formation of an LLDPE-carbocal biopolymer, via mixing, sieving, drying, and injection mounding. An analysis of the mechanical properties showed that higher carbocal content improved Young’s modulus but compromised the elongation at break. There were limited necking and plastic deformation.

#### 2.1.5. Chemical and Microbial Synthesis and Chemical Extraction

Biodegradable polymers are also generated by the activity of microorganisms such as Gram-negative and Gram-positive bacteria in the presence of carbon-rich materials such as agro-wastes. The bacterial production of the polymers is triggered by pH changes, limited availability of essential nutrients such as phosphorous and nitrogen [16], the composition and type of culture, and media [54]. The naturally occurring biopolymers act as biological storage systems or defense mechanisms. Microalgae are critical to the biological storage processes that result in the development of biopolymers, through biological carbon fixation via photosynthesis. The process culminates in the formation of branched polysaccharides. PHA is the leading bio-based biopolymer that is synthesized from microbes.

The synthesis of bio-based polymers from rice bran is catalyzed by the microbial activity of *Sinorhizobium meliloti* MTCC 100 bacteria. These bacteria are preferred compared to other species and synthetic methods because they do not pose a threat to the environment and generate significant quantities of agro-wastes [55]. The microbial synthesis method generated PHA, biomass, and exo-polysaccharides (EPS) at a rate of 3.63, 1.75, and 1.2 g/L, respectively [55]. The rate of production was augmented by the optimization of the incubation period and the addition of rice bran hydrolysate (RBH) at predefined intervals in the fermentation process. Fermentation time, temperature, and pH optimization experiments showed that optimal conditions for the synthesis of PHA biopolymer were neutral pH conditions, 30 °C, and 72 h. Even though the mechanical properties of the polymer were not measured, the FTIR spectra confirmed the presence of C=O, CH, and C-O-C [55]; these functional groups are associated with hydrogen bonding and advanced chemical bonding that help to predict the mechanical strength and the presence of specific functional groups such as cellulose and lignin [2]. Other microbes, such as white rot fungi, help in the natural de-lignification of agro-wastes [54].

Microbial synthesis methods have also proven effective in the production of poly b-hydroxybutyric acid (PHB)—a biodegradable and high strength PHA biopolymer [56]. The bacterial synthesis of the biopolymer is dependent on the availability of a carbon-rich precursor that is utilized by the bacteria as a source of food and energy. In contrast to other microbial synthesized biopolymers, PHB is suitable for high strength applications because it has mechanical properties that are nearly identical to petroleum-based biopolymers such as PP [56]. The primary constraint is the cost, which is nine-fold higher compared to other biopolymers. The cost is attributed to the market price of the carbon-rich starting materials. The cost-related factors have been resolved through the utilization of agro-wastes, such as wastes drawn from rice and jowar processing [56]. The utility of different strains of fungi and bacteria in agro-waste biopolymer synthesis shows that the quality of biopolymer produced was dependent on the types of cultures used and the media as shown in Table 3.

**Table 3:** Mechanical properties and optical properties of microbial synthesized starch films [57].

Type of Material	Optical Transmission (%)	Solubility (%)	Tensile Stress at Break (MPa)	Tensile Strain at Break (mm/mm)	Thickness (μm)	WVP (g.mm/Kpa. m^2^ h^1^)
Control	74.0 ± 3.10	15.19 ± 0.11	3.1 ± 0.39	0.35 ± 0.08	199 ± 26	1.9 ± 0.03
crystalline nanocellulose	64.4 ± 2.04	20.73 ± 0.05	3.3 ± 0.45	0.35 ± 0.04	183 ± 27	1.78 ± 0.06
Bacteriocin (from *P. acidilactici*)	63.2 ± 2.15	11.60 ± 0.20	2.85 ± 0.52	0.44 ± 0.02	195 ± 29	1.70 ± 0.06
Bacteriocin (from *E. faecium*)	62.2 ± 4.78	12.32 ± 0.21	3.04 ± 0.50	0.44 ± 0.05	198 ± 23	1.69 ± 0.03
BIN (bacteriocin from *P. acidilactici*)	53.9 ± 2.74	21.54 ± 0.51	4.33 ± 0.29	0.29 ± 0.03	187 ± 36	1.72 ± 0.04
BIN (bacteriocin from *E. faecium*)	52.1 ± 2.58	22.2 ± 0.48	5.24 ± 0.53	0.30 ± 0.02	187 ± 20	1.72 ± 0.04

WVP denotes—Water vapor permeability; BIN—Bacteriocins immobilized crystalline nanocellulose (BIN).

Unadulterated cultures were associated with higher volumetric productivity and high costs. In contrast, mixed cultures were affordable but resulted in poor yields [54]. Apart from the yield, the type of microbes predicted the rate of biodegradation, the rate of biodegradation oscillated between 46% and 63% [57]. The maximum rate of biodegradation was reported in bio-based polymers made from crystalline nanocellulose derived from agricultural sources. The presence of *E. faecium* resulted in the most significant reduction in the biodegradation rate but slightly higher tensile strength at break compared to the *P. acidilactici* species [57]. The microbes also impacted the optical properties and water vapor permeability (WVP). Biopolymers with minimal optical transmission % were ideal for greenhouse-related applications. Advanced methods involving genome sequencing have facilitated the synthesis of customized bacterial biopolymers such as PHB and PHA from recombinant *E. coli* and other microbes [58]. Advanced genetic methods have also facilitated the customization of the plant composition—the natural variability in plant cuticle distribution (raw materials for bio-based polymers) has been resolved by advanced breeding methods [59].

Chemical synthetic methods involve the treatment of agro-wastes/food wastes with acids and alkalis to extract the lignin and cellulose materials. The treatment processes form functional groups (C=O, and C-O-C, among others), which influence the mechanical properties of the biopolymer through the strength of the chemical bonds. On the downside, the chemical process generates furan derivatives, carboxylic acids, and lignin-derived phenols that inhibit the enzymatic activity, which is critical in the fermentation phase [54]. The negative impact of acids and alkalis on the fermentation process can be reversed by the incorporation of specific species of white-rot fungi such as *Ceriporiopsis subvermispora* in the pretreatment process [54]. The use of microbes in chemical synthesis does not negate the fact that synthetic chemicals are harmful to the environment and diminish the essence of using agricultural precursors in place of PE, PET, and PP.

## 3. Challenges in the Production of Biopolymers Polymers and Availability of Precursors

Kumar and Kumar [60] noted that available routes are characterized by an incompatibility between the hydrophilic water fibers and the hydrophobic polymer matrix, which is water repellent. The lack of compatibility leads to uneven dispersion and low mechanical strength. The inability to match the mechanical properties of non-biodegradable polymers limits the utilization of biodegradable polymers to applications that require low mechanical strength. The enhancement of the mechanical strength involves a tradeoff with biodegradability—the biodegradable materials have to be blended with polymers to enhance their mechanical strength [40]. Additionally, biological precursors such as cellulose acetate that yield high tensile strength (90 MPa) are not biodegradable [11,40]. The limited rate of biodegradation has been addressed in recent studies by replacing plant cellulose with bacterial cellulose, which has ideal water holding capacity and better biodegradation rates [1]. The unique properties of bacterial cellulose are associated with the ultrafine nano-fibrils in the 3D network structure. The variations in the mechanical properties of bio-based polymers and petrochemical-based polymers are presented in Table 4. The data show that biodegradable materials such as PHA [16] have limited tensile strength, elongation at break, and glass transition temperature compared to PET and PE [11].

**Table 4:** Comparative analysis of the mechanical properties of plant-based and petro-chemical based polymers [11].

Material	Tensile Strength (MPa)	Elongation at Break (%)	Glass Transition Temperature (°C)	Melting Temperature (°C)
Kraft paper	68	3		
Cellulose acetate	90	25	110	230
Corn starch	40	9	112	
PLA	59	2–7	55	165
PHA	15–50	1–800	12–3	100–175
PBS	34	560	32	114
PBAT	22	800	29	110
PEF	35–67	3–4	85	211
PTT	49	160	50	228
PE	15–30	1000	125	110–130
PP	36	400	13	176
PET	86	20	72	265
PS	30–60	1–5	100	–
PVC	52	35	18	200

Apart from the material-related shortcomings, the production of plastic materials is not economically viable compared to standard plastics. From an economic dimension, biodegradable plastics are not sustainable, considering that cost is a critical criterion in commercial applications. The data show that plant-based polymers such as PHA are four times more expensive relative to conventional polymers. The bio-based polymers cannot compete with standard plastics in the commercial market because consumers make purchase decisions based on the value of a product relative to the price [61]. The cost factor can be attributed to the economies of scale and limitations in viable technologies. Biodegradable polymers are produced at a smaller scale—99% of plastics (equivalent to about 335 million tons) produced for commercial applications are either non-biodegradable or partially biodegradable [13]. The lack of scalable technologies has also influenced the pricing of these materials.

### 3.1. Production and Market Sustainability of Bio-Based Polymers

The sustainability of bio-based plastics is dependent on an array of factors and criteria. The most critical are (i) the availability of commercially viable quantities of renewable feedstock and agricultural waste; (ii) scalable and facile production routes; (iii) cost and competition with synthetic polymers; and (iv) useful life and biodegradation/end of life treatment. Sustainable bio-based polymers should satisfy each of the four criteria. Even though empirical evidence suggests that agricultural waste is available in significant quantities, there has been an inadequate assessment of the availability of agricultural wastes that can help meet the global demand for bio-based polymers, especially in food packaging.

### 3.2. Availability of Commercially Viable Quantities of Renewable Feedstock and Agricultural Waste

FAO global estimates of the quantities of cereals, oilseeds, and pulses, roots and tubers, fruits, and vegetables that are lost each year globally suggest that 20–30% of fruits and vegetables are lost in farming (agriculture) and post-harvest phases across all continents. India and China have one of the highest rates of fruits and vegetable wastes, estimated at USD 484 million per year [19]. The financial losses are linked to the loss of 30–40% of fruit and vegetable produce. Cumulatively, India, UK, China, Mexico City, and Central de Abasto generate about 60 million tons of fruit and vegetable wastes each year [19]. Additional losses occur at the point of sale due to handling and poor consumption habits. Industrialized Asia had the lowest level of food waste [62]. The estimates are slightly lower compared to those reported by Alexander, Brown, Arneth, Finnigan, Moran, and Rounsevell [63]. The research estimated that wastes, losses, and inefficiencies in the supply system accounted for 44% of global food. The main question is whether the significant quantities of food that were discarded as waste are available in centralized locations. The availability of agricultural waste in centralized locations is critical, given the fast rates of biodegradation.

The FAO estimates on pulses and seeds that are lost in various agricultural supply chains and the level of wastage are lowest in industrialized Asia, North America and Oceania, and Europe [62]. The FAO estimates that 1.3 billion tons of food are wasted annually during farming and post-harvesting and agricultural processing. An EU-28 survey conducted between 2010 and 2016 estimated that 118 billion tons of agricultural wastes, co-products, and by-products (AWCB) were generated during that period [64]. Considering that 68% of this waste originated from fruits, cereals, and vegetables, the waste was a potential starting material for the production of bio-based polymers.

On the downside, the wastage occurs at the consumer or processor level, which limits the possibility that the agricultural waste would be collected and channeled towards the production of bio-based polymers. In general, the volume of waste does not predict the availability of agricultural waste for conversion into biopolymers because there are other competing applications such as composting [65], bio-fertilizer, and biogas production [12]. The Waste and Resources Action Programme (WRAP) and other social organizations across the EU are advocating for the responsible use of food to reduce the volume of food wastes—so far, these efforts have resulted in a 15% reduction in waste. If these efforts are sustained, food/agricultural waste will reduce significantly [65]. The challenge attributed to the absence of scalable and facile methods of synthesis is reviewed in the next section.

### 3.3. Scalable and Facile Production Routes

Current methods used in the production of bio-based polymers are not adequately scalable. The production of PHA from fruit peel discussed in Section 2.1.2 relies on west chemistry methods, whose level of efficiency is dependent on the physicochemical parameters [48]; the yield is not consistent. The production of bio-based plastics from selected sources requires extensive and advanced processing, which consequently impacts the cost of the material. For example, coffee grounds are highly hydrophilic and chemically incompatible with hydrophobic copolymers. The compatibility between these materials is augmented by the addition of coupling agents and compatibilizers [31]. Alternatively, the materials are subjected to thermal treatment under a vacuum environment to improve the hydrophobic properties. The limitations of coffee grounds show that not all agro-wastes are ideal precursors for the development of bio-based plastics.

The production of bio-based polymers from starch and biopolymer blends [18] relies on techniques that have limited commercial utility. Mater-Bi/Novamont, Minerv-PHATM, and Bio-Onhave, among other companies, adopted these methods in the production of bio-based polymers. However, the production capacity is unsatisfactory (97–560 kilotons) [34]. Another constraint is that optimal performance has been reported in biopolymer blends made of bioethanol, among other products that are not 100% biodegradable. The production of bioethanol competes with the human food supply chain and might increase the possibility of food insecurity; this is a common challenge for biofuels [66]. Emerging reports suggest that the production methods would be augmented by the development of new synthetic/production routes [29]. These promising methods are based on either pilot studies or applications that have not been proven on a commercial scale, such as the BBI-EU partnership [34]. Based on published evidence, there are limited facile and scalable methods for the production of bioplastics.

Diverse biopolymers can be developed from more than 100 types of available agro-waste. Additionally, diverse composites can be developed by integrating carbon fibers, cellulose, and natural anti-oxidants. Tabasum, Younas, Ansab, Majeed, Majeed, Noreen, Naeem, and Mahmood [42] documented 88 biopolymer types that can be developed from corn starch and natural, synthetic polymers. Each type of material had different characterization techniques and applications. From a production perspective, the diversity of the biopolymer materials that can be developed from available agro-wastes has a mixed impact. On the one hand, the mechanical, optical, and chemical properties can be customized through the addition of nanoparticles, and copolymers. On the other hand, the production of diverse biopolymer materials limits commercial applications because the imperfections in the new biopolymers cannot be resolved simultaneously. The limitations of modern synthetic routes could be the main reason why the production of bio-based polymers is unable to match synthetic polymers. The production of bio-based polymers was 2.1 million tons [13].

## 4. Applications of Agricultural Waste-Derived Biopolymers

The main applications of interest are in food packaging, construction, and agriculture [67]. The applications are influenced by the mechanical, physical, and chemical properties of the material. High strength applications in agriculture and construction require biopolymers with significant tensile strength/Young modulus. In contrast, flexibility/elongation at break is a key criterion in packaging applications. A compilation of different mechanical, physical, and chemical properties of commonly available agro-wastes showed that tamarind fruit fiber has the best mechanical properties (tensile strength of 1137–1360 MPa) [68], making it an ideal source of biopolymers for construction applications and a viable alternative to synthetic carbon. The data show that there is a relationship between the mechanical, physical, and chemical properties. The specific applications of the biopolymers in construction, food packaging, and automobiles are reviewed in Section 4.1, Section 4.2 and Section 4.3.

### 4.1. Application in Construction and Automobiles

**Figure 3:**
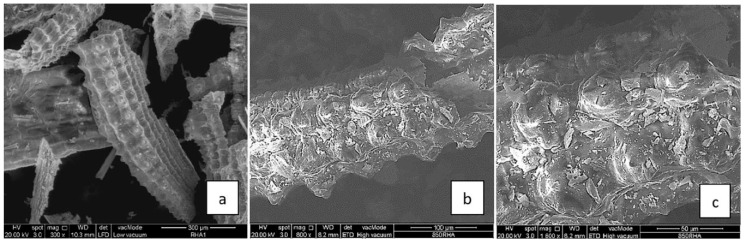
Surface and cross-sectional morphology of rice husk ash biopolymer before coating with CNFs, (**a**–**c**) for different scales [47] (Reproduced with permission from publisher).

**Figure 4:**
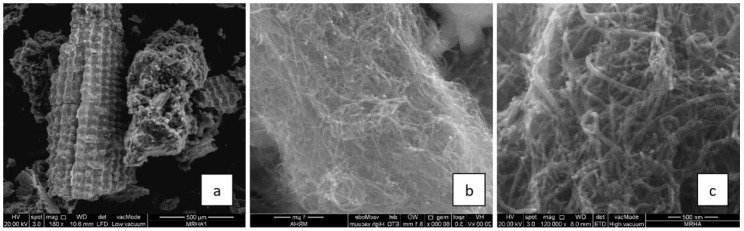
Surface and cross-sectional morphology of rice husk ash biopolymer after coating with CNFs, (**a**–**c**) for different scales [47] (Reproduced with permission from publisher).

The application of bio-based polymers in construction is influenced by the reinforcing materials such as carbon nanotubes (CNTs), carbon nanofibers (CNFs), nanocellulose [60], cellulose, lignin, hemicellulose, and α-cellulosic micro filler derived from agro-wastes [69]. The reinforcement of bio-based polymers is critical because the materials have high water permeability rates and are biodegradable. Advances in material science and nanotechnology have facilitated new applications in the construction sector. The reinforcement of bio-based polymers with carbon nanofibers (CNFs) and carbon nanotubes (CNTs) has increased the suitability of the rice-husk derived polymers in construction applications [47]. The changes in the surface and cross-sectional morphologies before and after coating with CNFs are illustrated in Figure 3 and Figure 4. CNTs are preferred because they have high tensile strength (7 GPa) and Young’s modulus (400 GPa) compared to biopolymers alone. The tensile strength of bio-based polymers such as PLA and PHA is below 100 MPa [11]. Additionally, the CNTs and CNFs have a wide aspect ratio and can be easily dispersed into the biopolymer-cement mixture.

The changes in surface and cross-sectional morphology after applying a coat of CNFs on rice husk ash. The presence of nano-scale fibers on the surface of the risk husk ash contributed to greater mechanical performance. In particular, a 15% modification of the risk husk ash resulted in a 187% improvement in the compressive strength and flexural strength after 28 days [47]. On the downside, the incorporation of the CNTs has undesirable effects on the concrete matrix—the higher van der Waal forces and specific surface energy in the composite lead to the agglomeration of the CNTs, a phenomenon that causes greater bridging effects and crack growth within the composite. The mechanical benefits afforded by the development of biopolymer-cement-CNT composite outweigh the risk of agglomeration and crack growth because agglomeration is reversible through ultrasonic and high-speed shear dispersion. Apart from CNFs and CNTs, the mechanical properties of rice husks can be modified by copolymer blending with PE and treatment with diazonium salt [70].

#### Polymer Matrix Composites (PMC) from Agro-Wastes

The application of biopolymers in construction applications is supported by the formation of polymer matrix composites from agro-wastes [50,71]. The main sources of the agro wastes are grape stalks, olive pits, and wet olive husks, sweet lime, and lemon peels [50,71]. The lime and lemon peels had an optimal tensile strength of 48 MPa. The mechanical strength of the PMCs derived from grape stalks, olive pits, and wet olive husks was attributed to the higher composition of cellulose, lignin and hemicellulose and the presence of basic and acidic groups, such as carboxyls, lactones and phenols, that are bonded through inter-and intra-molecular hydrogen bridge links [71]. On the downside, the reinforcement of the mechanical properties compromises the end of life treatment of the materials. PMCs and other composites that contain lignocellulose materials have higher thermal stability. The graph shows that the lignocellulose sample had a thermal degradation range of 362–694 °C. The improvement in thermal behavior is related to high carbon ratios [71]. Even though the lignocellulose materials are associated with high tensile strength, the utilization of the materials has significant environmental drawbacks, including incompatibility with commercially available biopolymers, poor wettability, and high rates of humidity absorption [31], which may compromise the integrity of the concrete structures.

Composites made of α-cellulosic micro fillers and epoxy matrixes have been used in construction applications to replace wood [69], and the replacement of interior metallic door panels in BMW and Mercedes-Benz branded automobiles. The α-cellulosic micro fillers are synthesized from agro-waste materials such as date seeds, Robusta coffee, coconut shells, wood, oil palm shells, walnut, hazelnut, and red coconut empty fruit bunch [69]. A comparative analysis of the performance of different materials shows that the coconut empty fruit bunch has comparable tensile strength as commercial cellulose—52 MPa [69]. The tensile strength is significantly higher relative to lignocellulosic and short fiber fillers made of oil palm shells, nuts, and banana, respectively. Additionally, the impact strength was higher compared to cellulose (0.85 versus 12.8 kJ/m^3^). The mechanical properties show that α-cellulosic micro fillers are suitable in high strength applications.

### 4.2. Applications in Agricultural Shade Nets (Anti-Insect Nets) and Mulching Films

Bio-based polymers made of cellulose, starch, polyhydroxyalkanoates (PHA), bio-polyethylene, and PLA are employed in the manufacturing of agricultural shade nets and mulching films [6,72,73]. The shade nets are vital in integrated pest management due to the toxicity of commercial pesticides—reducing the use of pesticides has ecological and economic benefits and better mechanical properties compared to the traditional LDPE films [73]. Additionally, the nets help to filter UV radiation, which is harmful to plant growth. The commercial application of these nets is influenced by tensile strength, mesh sizes [73], surface color, and chemical composition. Shade nets with high tensile strength have a longer useful life and are capable of withstanding meteorological hazards such as strong winds, sunlight, and hail [74].

### 4.3. Application in Food Packaging

Bio-based biopolymers that are effective in food packaging applications are PLA, sugar palm nano-fibrillated cellulose (SPNFCs), coffee grounds-PBAT composites, blueberry agro-industrial waste, and corn starch [33]. The choice of different agro-wastes in the production of food-packaging materials is based on the sustainability considerations listed in Section 6. The poor mechanical properties of PLA do not impact food packaging applications where tensile strength is not a critical factor. The low carbon footprint of PLA and other beneficial ecological effects show that PLA has the potential to replace polypropylene and polystyrene, among other non-biodegradable plastics used in packaging [34]. In general, the poor mechanical strength of unblended bio-based polymers coupled with the high rates of biodegradation and water permeability does not impact packaging applications in the food sector. According to Soares, Siqueira and Prabhakaram [36], bio-based polymers made through electrospinning/electrospray technology have found new applications in food packaging, tissue engineering, drug delivery systems, wound dressing, and enzyme immobilization. Coconut-fiber-based biopolymers have been used to develop handicrafts and gardening products [20].

The utility of agro-waste based packaging films has been enhanced by surface modification [38] using nano-fibrillated cellulose concentrations. Ilyas, Sapuana, Ibrahim, Abral, Ishak, Zainudin, Atikah, Nurazzia, Ansari, Syafri, Asrofi, Herlinam, and Jumaidin [38] noted that the modification of agro-waste biofilms made of sugar palm with nano-fibrillated cellulose concentrations resulted in a significant improvement in the physical, mechanical, and morphological properties. The mechanical properties of sugar palm nano-fibrillated cellulose (SPNFCs) was influenced by the cellulose content—an increase in the cellulose content from 0.1 to 1.0 wt% translated to a greater improvement in the mechanical strength [38]. The optimal ratio of SPNFCs was one—For modulus of elasticity and tensile strength. However, virgin sugar palm starch had the best performance in terms of elongation at break. The data show that higher concentrations of cellulose reduce the flexibility of the biopolymer.

The improvement in the mechanical characteristics can be linked to the microscopic changes that occur during the formation of the composite. The FESEM and TEM micrographs showed significant pore deformation, poor crack formation, and reinforcement of the matrix following the application of the starch coating. The FESEM and TEM data were augmented by the FTIR data, which confirmed the presence of C=O, C-O-C, and O-H functional groups based on the peaks that were observed at 995, 1335, and 1644 cm^−1^ [38]. In other studies, the presence of carboxyl acid groups, phenols, and lactones was associated with the formation of inter and intra-molecular hydrogen bridge links [68]. The presence of these functional groups confirmed that there was extensive hydrogen bonding, which translated to higher chemical bonding between the starch molecules and the SPNFCs. The XRD diffraction patterns showed that there was a considerable improvement in the relative crystallinity that was directly linked to the addition of the starch. In brief, the mechanical strength was linked to changes in the chemical composition that occurred during the development of the composite.

Modified coffee grounds have been used in the synthesis of bio-based films for packaging because virgin materials have limited hydrophobic properties that limit blending with synthetic polymers [31]. The material constraints of coffee grounds are resolved through the use of alternative reinforcing materials such as organo clay-based bio-nanocomposites, chitosan, carboxyl methylcellulose, polylactic acid, and lignocellulosic reinforced materials. There was also the formation of a PMC material from rosin/expanded rosin organoclay (ROC) and PLA and PBAT. The PMC synthetic route is an integral route to the development of commercial-grade biopolymers from agro-wastes. Coffee grounds are poor sources of biopolymers due to the hydrophobicity properties. The hydrophilic nature of coffee grounds impacts compatibility with hydrophobic polymers, limiting the bio-refinement related applications. The challenge has been resolved through the development of the polymer matrix, chemical or microbial treatment with coupling agents, and compatibilizers [31]. The compatibilizers can be replaced by bio-reinforcing agents, which improved the tensile strength and mechanical performance of the material relative to untreated materials. The stress–strain curve indicates that the treatment of virgin PBAT with a coffee grounds ratio of 10 yielded the best tensile strength performance at both 250 and 270 °C.

The modification of the biopolymer structure of blueberry powder and corn starch biopolymers via photobleaching contributed to the intelligent food packaging systems. The luminance values (surface color) of the biofilms were diminished by photo-bleaching and were ideal colorimetric indicators for packed food deterioration. The biofilms turned blue and red in acid and basic pH environments, respectively [33]. The deterioration of packed food is characterized by fermentation and an increase in the pH. The color changes can be discerned by the human eye. On the downside, the intelligent pH detection data are inconclusive because they are not correlated with the shelf life of foods containing different biomolecules such as proteins, lipids, salt, and sugar.

## 5. Useful Life and Biodegradation/End of Life (EoL) Treatment

The length of the useful life of bio-based polymers is dependent on the level of exposure to UV radiation, which induces photo-oxidation [4], heat-induced thermal degradation, risk of dissolution in water and mechanical strength in high strength applications. There are diverse options for the end of life treatment of biodegradable polymers; these include home decomposition, industrial composting, enzymatic depolymerization, catalytic recycling, chemical recycling, mechanical recycling, and anaerobic digestion. The choice of each method of EoL treatment is informed by the type of precursor. For example, PLA is primarily recycled via mechanical recycling, chemical recycling, or industrial composting [34]. The options available have helped to mitigate the risk of global warming and carbon emissions. However, these methods are not 100% recyclable. A biodegradation rate of between 60% and 80% has been reported in previous studies [75,76]. Cellulose-based biopolymers achieved a 70% biodegradation rate after 350 days [75], while a similar rate of biodegradation was reported after five months in agro-based composite materials [76]. The rate of biodegradation is also contingent on the microbes used in microbial synthesis. Biopolymers synthesized using *P. acidilactici,* and *E. faecium* had the lowest biodegradation rates (<50%) [57]. The addition of reinforcing agents resulted in a further decline in the rate of biodegradation. The limited rates of biodegradation raise fundamental questions on whether the materials classified as “biodegradables” are truly biodegradable or only compostable. The ISO 14,855 standards indicates that a material satisfies the biodegradability criteria if 90% of the initial mass is lost within 6 months at 59 °C [77]. Additional provisions under ASTM D5338 indicate that biopolymer blends are biodegradable if they achieve 90% loss in mass within 180 days. The acceptable rate of mass loss of homopolymers after 180 days is 60% [78]. A material that does not satisfy the biodegradable criteria can be categorized as compostable because compostable plastics are biodegradable but biodegradable plastics might not be compostable [79]; mass losses define the distinction between biodegradation and compostability.

The main concern is that the rate of biodegradation varies widely depending on the environment (marine, soil and freshwater). Other concerns include limited data on biodegradable polymers and polymers derived from agricultural wastes that satisfied these criteria. Despite the paucity of information, it is evident that cellulose-based biopolymers and materials made by *P. acidilactici,* and *E. faecium* [57,75] did not meet the ISO criteria for biodegradation. The risk to the environment is not eliminated even in 100% biodegradable polymers, because nano-scale materials originating from the degraded polymers have the potential to trigger water and air pollution [75].

### Compostability of PLA and PHA Biopolymers

Experimental data do not provide conclusive evidence on whether PLA and PHA biopolymers are fully biodegradable or only compostable. Based on the ISO definition [77], both PLA and PHA polymers are not 100% biodegradable. Recent experiments noted that PHA/PLA materials achieved a 68–72% mineralization in 90 days [80]; there is no further evidence on whether the rates improved in the post-90 day period or whether the rate of degradation stagnated. Even though PHA/PLA biopolymers do not satisfy the biodegradability criteria, Emadian, Onay and Demirel classified these materials as biodegradable [79]. In brief, there is no consensus among researchers on compostability and biodegradability of biopolymers, biodegradable polymers, and polymers from renewable agricultural waste.

## 6. Cost, Consumer Attitudes and Competition with Synthetic Polymers

The cost of bio-based polymers has traditionally been a critical impediment in commercial applications owing to shortcomings in production methods. The development of microbial synthesis routes (anaerobic and aerobic processing, in mixed cultures and media) and availability of cheap carbon-rich precursors has facilitated the development of affordable PHA for medical and other commercial applications [81]. On the downside, mixed cultures are affordable but result in poor yields and volumetric productivity [54]. The issue of cost and competition is also influenced by consumer purchase preferences and worldview towards sustainable products. Previous market research studies affirmed that consumers are not inclined to change their purchase decisions based on ecological factors alone. The product must have comparable or similar performance to the replaced product. A majority of the new millennial consumers express support for environmentally conscious production, but seldom initiate purchase decisions based on these factors [82]. The elusive green consumer phenomenon is paradoxical; it also underscores the economic risks associated with the capital-intensive production of bio-based polymers. In brief, the sustainability domain of bio-based polymers is not adequate to facilitate a market-wide transition. The products must be value-adding. A unique value proposition for biofilms used in packaging is intelligent packaging. New synthetic routes have led to the production of biofilms that can detect the degradation of food through calorimetric pH changes [33]. Another unique value proposition is ethylene absorption, protection against UV radiation, elimination of water vapor, and enhanced anti-microbial activity against common bacteria such as *E. coli, Bacillus subtilis,* and *Listeria monocytogenes*. Synthetic plastics lack these properties.

## 7. Conclusions

This review article yielded new knowledge on the production of biopolymers, biodegradable polymers, and polymers from renewable agricultural waste sources such as grape and tomato pomace, green tea extracts, essential oils, and curcumin, coconut shells, vegetable waste, rice husks, fruit peels, grapefruit seed extract, waste vegetables, maize and wheat starch, and municipal agro wastes. Sustainability is a primary criterion that influences the choice of the precursor (the type of agro-wastes). The production of biopolymers requires commercially viable quantities of agro-waste—a key challenge considering that the wastes occur at the retail and household levels, and there is no mechanism for sorting and disposal of the wastes. Additionally, there is a global variability in the availability of agro-wastes, a factor that influenced the mechanical and optical properties of the polymers developed. Fruit peels and coconut shells are common in fruit growing regions in tropical and subtropical areas, and coastal areas, respectively. Grape pomace waste is available in regions with grapevines such as Italy. The variations in the availability of waste impact the rate of production. Another constraint is the lack of facile and scalable synthetic routes. New and novel methods are based on laboratory models or experiments, which have not been applied on a commercial scale. Commercial methods include copolymer blending and chemical synthesis; these methods led to the formation of biofilms and bio-plastics, which are not 100% biodegradable. The reinforcement of the mechanical properties involves a trade-off with the elongation at break, thermal degradation ability (at the end of life treatment), and ecological impact, including carbon footprint, and eco-toxicity.

The adverse impact of chemical additives, stabilizers, and photo-initiators has been ameliorated through the development of bio-based anti-oxidant additives made from agro-wastes such as mango kernel extracts, green tea extracts, essential oils, proto-catechuic acid, grapefruit seed extract. The limited synthetic methods available have impacted the costs and the ability of the bio-based plastics to favorably compete with synthetic polymers in the market. The cost factor partly explains why the global market share of bio-based plastics is below 1%. Other emerging concerns include the end of life treatment and useful life—natural bio-based polymers are susceptible to water attack and lack appropriate mechanical strength. Surface doping, blending with commercial polymers and the formation of polymer matrix composites improve the mechanical strength and reduce the rate of biodegradation. The current state of research and development in the production of bio-based plastics predicts the future of bio-based plastics and contribution to global sustainability. The progress made in the production of bio-based films through electrospinning/electrospray technology, nano fibrillated cellulose concentrations, and reinforcement with cellulose has contributed to the demand for biofilms in packaging. The utility of biopolymers in construction and agricultural applications is contingent on the availability of synthetic methods that balance between the tensile and flexural strength, biodegradation, and ecological impact.

## Figures and Tables

**Table 1:** Physical properties of different types of biopolymers [18].

Property	Type of Biopolymer						
	PLA	l-PLA	dl-PLA	PGA	PCL	PHB	Starch
Density(kg/m^3^)	1210	1240	1250	1500	1110	1180	
Tensile strength (MPa)	21	15.5	27.6	60	20.7	40	5.0
Young’s Modulus (GPa)	0.35	2.7	1	6	0.21	3.5	0.125
Elongation (%)	2.5	3	2	1.5	300	5	31
Glass transition temperature (°C)	45	55	50	35	−60	5	
Melting temperature (°C)	150	170	am	220	58	168	

**Table 2:** Chemical composition of common forms of agricultural waste [39].

Agro-Industrial Wastes	Chemical Composition (% w/w)					
	Cellulose	Hemicellulose	Lignin	Ash (%)	Total Solids (%)	Moisture (%)
Sugarcane bagasse	30.2	56.7	13.4	1.9	91.66	4.8
Rice straw	39.2	23.5	36.1	12.4	98.62	6.58
Corn stalks	61.2	19.3	6.9	10.8	97.78	6.40
Sawdust	45.1	28.1	24.2	1.2	98.54	1.12
Sugar beet waste	26.3	18.5	2.5	4.8	87.5	12.4
Barley straw	33.8	21.9	13.8	11	_	_
Cotton stalks	58.5	14.4	21.5	9.98	_	7.45
Oat straw	39.4	27.1	17.5	8	_	_
Soya stalks	34.5	24.8	19.8	10.39	_	11.84
Sunflower stalks	42.1	29.7	13.4	11.17	_	_
Wheat straw	32.9	24.0	8.9	6.7	95.6	7
